# Soluble Receptor for Advanced Glycation End-Product (sRAGE)/Pentosidine Ratio: A Potential Risk Factor Determinant for Type 2 Diabetic Retinopathy

**DOI:** 10.3390/ijms14047480

**Published:** 2013-04-03

**Authors:** Zhi Xiang Ng, Kek Heng Chua, Tajunisah Iqbal, Umah Rani Kuppusamy

**Affiliations:** 1 Department of Biomedical Science, Faculty of Medicine, University of Malaya, Kuala Lumpur 50603, Malaysia; E-Mails: ngzx_86@yahoo.com (Z.X.N.); khchua@um.edu.my (K.H.C.); 2 Department of Ophthalmology, Faculty of Medicine, University of Malaya, Kuala Lumpur 50603, Malaysia; E-Mail: tajun69@yahoo.com

**Keywords:** advanced oxidation protein product, antioxidant enzymes, diabetic retinopathy, oxidative stress, pentosidine, risk factor, soluble receptor for advanced glycation end-product

## Abstract

This study aims to investigate potential diabetic retinopathy (DR) risk factors by evaluating the circulating levels of pentosidine, soluble receptor for advanced glycation end-product (sRAGE), advanced oxidation protein product (AOPP) as well as glutathione peroxidase (GPx) and superoxide dismutase (SOD) activities in DR patients. A total of 235 healthy controls, 171 type 2 diabetic without retinopathy (DNR) and 200 diabetic retinopathy (DR) patients were recruited. Plasma was extracted for the estimation of pentosidine, sRAGE, AOPP levels and GPx activity whereas peripheral blood mononuclear cells were disrupted for SOD activity measurement. DNR and DR patients showed significantly higher levels of plasma pentosidine, sRAGE and AOPP but lower GPx and SOD activities when compared to healthy controls. The sRAGE/pentosidine ratio in DR patients was significantly lower than the ratio detected in DNR patients. Proliferative DR patients had significantly higher levels of plasma pentosidine, sRAGE, AOPP and sRAGE/pentosidine ratio than non-proliferative DR patients. High HbA_1c_ level, long duration of diabetes and low sRAGE/pentosidine ratio were determined as the risk factors for DR. This study suggests that sRAGE/pentosidine ratio could serve as a risk factor determinant for type 2 DR as it has a positive correlation with the severity of DR.

## 1. Introduction

Diabetes mellitus (DM) is one of the top four most conspicuous non communicable diseases that lead to death worldwide [1]. The International Diabetes Federation predicts that South East Asia region would have the highest prevalence of DM in 2025 [2] mainly due to the population growth and increase in the rate of obesity. The worldwide rise in DM prevalence will inevitably be accompanied by the increased incidence of irreversible DM associated complications which includes diabetic retinopathy (DR). DR is the greatest fear among DM related ocular complications as it can lead to blindness and disability [3]. The prevalence of DR among type 2 DM patients in Malaysia is on the rise, with 10% after 5 years and 42.9% after 10 years of diagnosis [4].

Oxidative stress is an acknowledged pathogenic mechanism in complications of DM. Hyperglycemia stimulates the increased production of reactive oxygen species through several mechanisms including glucose auto-oxidation, redox imbalance, advanced glycation end products (AGE)-receptor interaction, oxidative phosphorylation, lipooxygenase, cytochrome P450 monooxygenase and nitric oxide synthase [5,6]. The production of reactive oxygen species leads to the depletion of both enzymatic and non-enzymatic antioxidants which inevitably results in cellular damage [7]. Numerous studies have shown that treatment with antioxidants ameliorates complications of DM [8]. The DM environment causes proteins to undergo sequential non-enzymatic glycation with reducing sugar to form AGE [9]. Pentosidine is one of the well characterized AGEs. The formation of AGE is accompanied by oxidative, radical-generating reactions. There is substantial evidence to support that the binding of AGE to its receptor (RAGE) is involved in the development of microvascular complications [9,10]. Soluble RAGE (sRAGE), a RAGE isoform lacking the transmembrane domain, is a recently discovered inhibitor of AGE-RAGE mediated pathological effects [11]. It was shown to confer protection on blood vessels against AGE-RAGE mediated microvascular damage in DM [12,13]. However, the association between sRAGE and other oxidative-glycation biomarkers such as pentosidine and advanced oxidation protein product (AOPP) in DR patients are still unclear.

This study was aimed to investigate potential clinical risk factors for DR by evaluating the relationship between selected parameters in the oxidative system, mainly the circulating pentosidine, sRAGE, AOPP, glutathione peroxidase (GPx) and superoxide dismutase (SOD) in DR.

## 2. Results

The demographic data of healthy controls, DNR, DR, NPDR and PDR patients are stated in Table 1. The patients (DNR and DR) had significantly (*p* < 0.05) higher levels of glycated haemoglobin (HbA_1c_), total cholesterol, LDL-C and systolic blood pressure (SBP), lower diastolic blood pressure (DBP) and HDL-C/LDL-C ratio when compared to the healthy controls. Besides, the number of hypertensive subjects in the patient groups was significantly higher than in the healthy controls. Comparisons between DNR and DR patients showed significant differences (*p* < 0.05) in the levels of HbA_1c_ and total cholesterol, estimated glomerular filtration rate, duration of DM and the number of smokers. No significant difference was found for most of the parameters in NPDR and PDR patients except for age, HDL-C, triglycerides, SBP and retinopathy duration. PDR patients had significantly higher triglycerides level and SBP, longer duration of DR but had significantly lower HDL-C level and number of subjects who received antihyperglycemic treatment when compared to NPDR patients.

**Figure 1:**
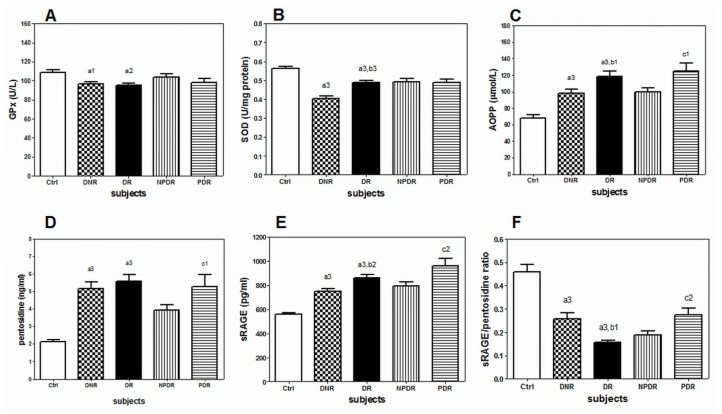
The measured biochemical parameters in Ctrl (*n* = 235), DNR (*n* = 171), DR (*n* = 200), NPDR (*n* = 125) and DR (*n* = 75) patients. Data were expressed as mean ± SD. AOPP = advanced oxidation protein product; Ctrl = healthy controls; DNR = diabetic non-retinopathy; DR = diabetic retinopathy; GPx = glutathione peroxidase; SOD = superoxide dismutase; sRAGE = soluble form of RAGE. ^a1^*p* < 0.05, ^a2^*P* < 0.01, ^a3^*p* < 0.001 *versus* healthy controls; ^b1^*p* < 0.05, ^b2^*p* < 0.01, ^b3^*p* < 0.001 *versus* DNR; ^c1^*p* < 0.05, ^c2^*p* < 0.01 *versus* NPDR.

In this study, the patients (DNR and DR) showed significant elevation of plasma pentosidine, sRAGE and AOPP levels as compared to the healthy controls (Figure 1). However, significantly lower sRAGE/pentosidine ratio, plasma GPx and SOD activities in the PBMC were present in the patients (DNR and DR). Interestingly, DR patients showed significantly higher levels of plasma sRAGE and AOPP but lower sRAGE/pentosidine ratio when compared to DNR patients. Comparison between NPDR and PDR patients showed that PDR patients had significantly higher levels of plasma pentosidine, sRAGE, AOPP and sRAGE/pentosidine ratio (Figure 1).

The correlation results shown in this study (Tables 2 and 3) were weak (*r* < 0.5) but significant (*p* < 0.05). The risk factor for DR among type 2 DM patients was investigated using the logistic regression model. Parameters such as age, smoker, HbA_1c_ level_,_ DM duration, total cholesterol level, LDL-C level, SOD activity and sRAGE/pentosidine ratio were significant determinants for DR (Table 4). However, after adjusting for age, gender and metabolic risk factors (BMI, HbA_1c_, duration of DM), HbA_1c_ level (OR = 1.20, 95% CI = 1.00–1.44, *p* = 0.046), duration of DM (OR = 1.08, 95% CI = 1.04–1.13, *p* = 0.000) and sRAGE/pentosidine ratio (OR = 0.08, 95% CI = 0.01–0.98, *p* = 0.048) still remained significant.

## 3. Discussion

Soluble RAGE (sRAGE), a RAGE isoform lacking the transmembrane domain, is a recently discovered naturally occurring inhibitor of AGE-RAGE mediated pathological effects [11]. Conflicting results on the sRAGE level in DM patients have been reported previously [14,15]. In this study, antihypertensive treatment did not significantly affect the sRAGE level in the patients (data not shown) which is in accordance with a previous report [16]. Therefore, it is unlikely that such treatment could confound the present results although certain antihypertensive medications have been shown to modulate sRAGE level in human subjects [17]. Our study shows that sRAGE level and DM duration in DNR patients were positively correlated (Table 2). The increased plasma sRAGE level in DNR and DR patients could be viewed as a protective reaction to counterbalance, at least partly, the elevated plasma pentosidine level. However, the regulatory mechanisms that are responsible for the increased sRAGE are unknown and remain to be elucidated. Due to the presence of intracellular glycation in DM and the fact that pentosidine is not a major ligand for RAGE [17], the increased circulating level of plasma sRAGE would not likely reduce the rate of DR progression in type 2 DM patients.

Interestingly, DR patients had a significantly lower sRAGE/pentosidine ratio than DNR patients. It should be noted that sRAGE level was markedly elevated in DR patients whereas an increase in pentosidine level was observed in all DM patients, independent of retinopathy complication. PDR patients with longer duration of retinopathy had significantly higher sRAGE/pentosidine ratio than NPDR patients and sRAGE/pentosidine ratio was positively correlated with retinopathy duration (Table 3). Thus, high sRAGE/pentosidine ratio among the DR patients may be used to predict the severity of DR. With the finding of sRAGE/pentosidine ratio as a significant determinant for DR (Table 4), this study suggests that sRAGE/pentosidine ratio could serve as a risk factor for DR among type 2 DM patients. In contrast to a previous report [18], pentosidine level was not significantly different between DR and DNR patients in this study. This could be explained by the variation in patient populations [19]. Our patients were of type 2 DM, older and had higher HbA_1c_ level when compared to the patients recruited in a previous study [18]. The patient groups (DNR and DR) in this study consisted of a significant number of Indian subjects when compared to the healthy controls as it was not possible to obtain significant healthy Indian volunteers in the age-group of interest. Nevertheless, the measured parameters in the healthy volunteers from various ethnic groups did not show significant differences.

Oxidative stress is widely recognized as the key component in the pathogenesis of diabetic complications. Human bodies are constantly protected against excessive oxidative stress by a complex set of enzymatic and non-enzymatic antioxidant systems. Both enzymatic antioxidants superoxide dismutase (SOD) and glutathione peroxidase (GPx) are involved in the metabolism of superoxide and hydrogen peroxide respectively [20]. The significant decrease of GPx and SOD activity in DNR and DR patients found in this study provides further evidence on the imbalance of antioxidant system in subjects with DM complications [21]. Numerous stable end products of oxidative stress have been identified and these include the AOPP. AOPP also serves as a biomarker for inflammation [22]. The elevated levels of plasma AOPP and pentosidine in DNR and DR patients as well as the significant positive correlation between pentosidine and HbA_1c_, DM duration in DR patients (Table 3) support the presence of oxidative stress in DR patients.

In addition, the negative correlations found between enzymatic antioxidant activity (GPx and SOD) and circulating oxidative-glycation products (AOPP and pentosidine) in both DNR and DR patients in this study (Tables 2 and 3) confirmed the inverse relationship between the antioxidant status and hyperglycemia-dependent cellular changes in patients with type 2 DM. Other than HbA_1c_ level, AOPP was also positively correlated with total cholesterol level in DNR patients and triglycerides level in both the patient groups (Tables 2 and 3). This finding supports the fact that hyperglycemia is not the only causal factor for exacerbating oxidative damage in DR patients. The generation of superoxide via mitochondrial electron transport chain [19] and the activation of NADPH-oxidase in monocytes triggered by AOPP [23] could also contribute to oxidative damage in DR.

N-carboxymethyl-lysine (CML), an oxidatively formed AGE has been previously shown to be present both as a regular constituent in non-DM retinas and in neuroglial vasculature of DM patients [24] and it has been shown to be associated with proliferative DR and macular oedema [25]. Meanwhile pentosidine is a non-oxidatively formed AGE, which has specificity to microvasculature and the entire retina of DM patients while co-localizing with the expression of RAGE [24]. The accumulation of AGE in DR patients could exert its toxic effect through several mechanisms such as by modifying the protein’s tertiary structure through cross-linking, impairing receptor recognition and altering enzymatic activity [19]. The sustained AGE-RAGE interaction in DR patients could activate pro-inflammatory pathways by inducing the expression of cytokines and adhesive molecules [26], all of which could affect the blood-retinal barrier. AOPP shares common biological effect exerted by AGE, including interaction with RAGE [26] which ultimately leads to neovascularisation that could result in DR.

## 4. Experimental Section

### 4.1. Ethic Statement

Written informed consent was obtained from each subject prior to blood collection. The study was performed in adherence to the principles of the Declaration of Helsinki and approved by the Medical Ethics Review Committee of University Malaya Medical Centre, Malaysia (Institutional Review Board Number: 744.12).

### 4.2. Study Population

The study subjects were patients with type 2 DM referred to the Ophthalmology clinic between September 2009 and December 2011 at University Malaya Medical Centre (UMMC), Malaysia. DM was diagnosed according to the World Health Organization criteria. Type 1 DM and type 2 DM patients with complications other than retinopathy were excluded from the study. The blood creatinine level and estimated glomerular filtration rate in all the patients were examined to exclude those with possible nephropathy complication. Subjects with previous history of chronic inflammatory diseases, who were on anti-inflammatory drug treatment and antioxidant supplements were also excluded as these factors would affect the oxidative stress related parameters. A total of 371 unrelated Type 2 DM patients [171 patients without retinopathy (DNR) and 200 with retinopathy (DR)] (210 men, 161 women) aged 58.2 ± 9.7 years (mean ± SD; range, 40 to 78 years), were recruited. Detailed medical and ophthalmologic histories as well as socio-demographic factors and lifestyle variables of each patient were obtained.

All the patients recruited in this study underwent a complete eye examination that included dilated retinal examination and 7-field stereoscopic Diabetic Retinopathy Study retinal photography [27]. For DR patients, the color fundus photographs were graded for DR severity in a masked fashion by two independent ophthalmologists at University of Malaya Eye Research Centre, Kuala Lumpur. The modified Early Treatment of Diabetic Retinopathy Study Airlie House classification of DR was used to grade the retinopathy into the following categories: mild non-proliferative retinopathy (mild NPDR), moderate non-proliferative retinopathy (moderate NPDR), severe non-proliferative retinopathy (severe NPDR) and proliferative retinopathy (PDR) [28,29]. Among the DR patients, 26 had mild NPDR, 85 moderate NPDR, 14 severe NPDR and 75 PDR.

The healthy controls were blood-donor volunteers, consisting of 235 subjects (134 men, 101 women) aged 57.1 ± 4.1 years (mean ± SD; range, 45 to 65 years). They were recruited from multiple blood donation campaigns held between January 2010 and October 2010 in Malaysia. Their blood profiles were examined by the attending doctors through clinical and biochemical methods.

### 4.3. Sample Collection and Preparation

Six mL of blood was collected from patients and control subjects. Three mL of blood was used for glycated haemoglobin (HbA_1c_), total cholesterol, high-density lipoprotein (HDL-C), low-density lipoprotein (LDL-C), triglycerides, creatinine, alanine amino transferase (ALT) and aspartate amino transferase (AST) measurement at the Clinical Diagnostic Laboratory, University Malaya Medical Centre. The whole blood samples were centrifuged for 15 min at 1000× *g*. The plasma was extracted and stored at −80 °C for pentosidine enzyme-linked immunosorbent assay (ELISA), sRAGE ELISA, GPx and AOPP assays. Subsequently, the cell sediments of EDTA blood tubes were reconstituted with isotonic phosphate buffer saline solution. The peripheral blood mononuclear cells (PBMC) were isolated as previously described [30], based on density gradient centrifugation method. PBMCs (2 × 10^6^ cells) were disrupted by freeze-thaw method. The cytosolic extract was centrifuged at 14,000× *g* for 10 min at 4 °C and the supernatant was used for SOD assay as well as total protein concentration determination as previously described [31]. Analyses of all samples were performed within 1 month of collection.

### 4.4. Measurement of Plasma Pentosidine

Plasma pentosidine was measured with sandwich ELISA standard kit (USCNK Life Science Inc., Wu Han, China), according to the manufacturer’s protocol. The plate was coated with monoclonal antibody against human pentosidine and a polyclonal antibody was used for detection. Mean minimal detectable dose of pentosidine was 0.087 ng/mL. Results were expressed as ng/mL. The inter-assay coefficient of variation was 5.1%.

### 4.5. Measurement of Plasma sRAGE

Plasma sRAGE was measured with sandwich ELISA standard kit (Biovendor Laboratorni Medicina akciová společnost, Brno, Řečkovice, Czech Republic), according to the manufacturer’s protocol. The plate was coated with monoclonal antibody against human sRAGE and a polyclonal antibody was used for detection. Mean minimal detectable dose of sRAGE was 19.2 pg/mL. Results were expressed as pg/mL. The inter-assay coefficient of variation was 3.6%.

### 4.6. Measurement of Plasma AOPP

The plasma level of AOPP was quantitatively determined by using colorimetric method as described previously [32]. Chloramine-T was used as the standard. Results were expressed as μmol/L. The inter-assay coefficient of variation was 4.6%.

### 4.7. Measurement of Plasma GPx Activity

The plasma GPx activity was determined using standard kit (EnzyChrom™ GPx, Bioassay Systems, Hayward, CA, USA), according to the manufacturer’s protocol. The assay is based on the oxidation of NADPH in an enzyme coupled reaction. The linear detection range was 12–300 U/L GPx activity. Results were expressed as U/L. The inter-assay coefficient of variation was 5.7%.

### 4.8. Measurement of PBMC SOD Activity

The SOD activity in PBMC was measured using a standard kit (Cayman Chemical Company, Ann Arbor, MI, USA), according to the manufacturer’s protocol. The assay kit utilizes a tetrazolium salt for detection of superoxide radicals generated by xanthine oxidase and hypoxanthine. It can be used to detect all 3 types of SOD (Cu/Zn, Mn, and FeSOD), with dynamic range of 0.025–0.25 units/mL SOD. Cytosol protein concentration was determined as previously described [31] and the SOD activity was expressed as U/mg. The inter-assay coefficient of variation was 4.5%.

### 4.9. Statistical Analysis

The continuous variables were checked for normality prior to the statistical analysis. Chi-Square test with one degree of freedom (for dichotomous variables) and unpaired t-test (for continuous variables) were used for the evaluation of differences between groups. Comparison of subgroups was performed with ONE-WAY ANOVA and post hoc test of Tukey’s multiple comparisons. Association between parameters was determined by Pearson’s correlation coefficient (*r*) with Bonferroni correction. Logistic regression model was used to estimate the odds ratios (ORs) and 95% confidence intervals (CIs) for each risk factor for DR among type 2 DM patients. Statistical significance was set at *p* < 0.05. All data were analyzed using IBM statistical package for the social sciences (IBM-SPSS) version 18.0 for Windows (IBM Co., New York, NY, USA).

## 5. Conclusions

In conclusion, this study suggests that sRAGE/pentosidine ratio could serve as a risk factor determinant for DR as it has a positive correlation with the severity of type 2 DR. This study also provides further evidences for the presence of oxidative stress (decreased GPx, SOD activities and increased accumulation of AOPP and pentosidine) in DR patients. The interaction between sRAGE and other biomarkers in microvascular injury as well as the pathophysiologic mechanisms involved deserve further investigation.

## Figures and Tables

**Table 1:** Demographic profiles of the healthy controls, DNR, DR, NPDR and PDR patients.

Demography	Ctrl (*n* = 235)	DNR (*n* = 171)	DR (*n* = 200)	NPDR (*n* = 125)	PDR (*n* = 75)
Gender (male/female)	134/101	100/71	110/90	67/58	43/32
Races (Malay/Chinese/Indian) (n)	106/90/39	63/28/80 a	70/47/83 a	40/29/56	30/18/27
Age (years)	57.1 ± 4.1	59.2 ± 9.6	57.2 ± 9.8	59.0 ± 9.9	53.1 ± 8.8 c
BMI (kg/m^2^)	25.6 ± 4.8	27.2 ± 4.4	26.3 ± 5.0	26.2 ± 4.5	26.6 ± 5.9
HbA_1c_ (%)	5.6 ± 0.4	7.9 ± 1.8 a	8.9 ± 2.1 a,b	9.1 ± 2.3	8.5 ± 1.8
Total cholesterol (mmol/L)	3.8 ± 0.6	4.5 ± 1.0 a	4.8 ± 1.5 a,b	4.8 ± 1.4	4.9 ± 1.5
Triglyceride (mmol/L)	1.8 ± 1.3	1.6 ± 0.7	1.7 ± 1.0	1.6 ± 0.9	2.0 ± 1.1 c
HDL-C (mmol/L)	1.0 ± 0.3	1.2 ± 0.3 a	1.2 ± 0.3 a	1.3 ± 0.3	1.1 ± 0.3 c
LDL-C (mmol/L)	2.1 ± 0.5	2.5 ± 0.9 a	2.8 ± 1.2 a	2.8 ± 1.2	2.9 ± 1.3
HDL-C/LDL-C ratio	0.6 ± 0.2	0.5 ± 0.2 a	0.5 ± 0.2 a	0.5 ± 0.2	0.4 ± 0.2
SBP (mmHg)	124.0 ± 8.0	136.5 ± 19.5 a	139.3 ± 22.4 a	136.6 ± 21.4	146.6 ± 23.0 c
DBP (mmHg)	83.0 ± 7.0	79.0 ± 10.5 a	78.4 ± 13.1 a	77.2 ± 11.9	81.6 ± 15.6
ALT (IU/L)	–	37.8 ± 17.5	36.8 ± 24.6	38.1 ± 27.0	33.9 ± 18.3
AST (IU/L)	–	22.0 ± 14.0	22.8 ± 16.4	22.7 ± 17.2	23.0 ± 15.0
Creatinine (μmol/L)	–	94.9 ± 13.4	98.1 ± 12.8	90.0 ± 15.4	100.1 ± 13.6
eGFR (ml/min/1.73m^2^)	–	82.6 ± 9.6	90.2 ± 10.4 b	92.2 ± 7.3	88.3 ± 12.7
Diabetes duration (years)	–	10.4 ± 7.9	15.7 ± 9.1 b	16.3 ± 9.4	14.6 ± 8.2
Retinopathy duration d (years)	–	–	5.0 ± 3.6	3.4 ± 2.0	7.4 ± 4.3 c
Current smoker (n)	43	29	13 a,b	7	6
Hypertension (n)	11	104 a	119 a	80	39
Antihyperglycemic treatment e (n)	0	107 a	130^a^	93	37 c
Duration of antihyperglycemic treatment (years)	–	9.5 ± 5.5	11.5 ± 7.5	9.9 ± 3.6	11.0 ± 5.2
Antihypertensive treatment f (n)	0	104 a	119 a	79	40
Duration of antihypertensive treatment (years)	–	7.0 ± 3.5	8.5 ± 4.0	7.7 ± 2.8	8.3 ± 3.5

Notes: Data were expressed as mean ± SD unless otherwise indicated. Dichotomous variables are given in absolute numbers.

a *p* < 0.05 *versus* healthy control;

b *p* < 0.05 *versus* DNR;

c *p* < 0.05 *versus* NPDR;

d Period derived from the time when retinopathy complication was first diagnosed in the patients;

e Oral medications and insulin;

f Inclusive of angiotensin-converting enzyme inhibitors, angiotensin II type I receptor antagonists, calcium channel blockers and diuretics; ALT = alanine aminotransferase; AST = aspartate aminotransferase; BMI = body mass index; Ctrl = healthy controls; DBP= diastolic blood pressure; DNR = diabetic non-retinopathy; DR = diabetic retinopathy; eGFR = estimated glomerular filtration rate; HbA_1c_ = glycated haemoglobin; HDL-C = high-density lipoprotein cholesterol; LDL-C = low-density lipoprotein cholesterol; NPDR = non-proliferative diabetic retinopathy; PDR = proliferative diabetic retinopathy; SBP = systolic blood pressure.

**Table 2:** Linear correlation analysis between AOPP, pentosidine, sRAGE levels and several biochemical parameters in DNR patients (*n* = 171).

Variables	AOPP (μmol/L)		Pentosidine (ng/mL)		sRAGE (pg/mL)	
		
	*r*	*p* value	*r*	*p* value	*r*	*p* value
HbA_1c_ (%)	0.20	0.017	0.07	NS	0.03	NS
Total cholesterol (mmol/L)	0.19	0.026	−0.05	NS	−0.17	0.047
Triglycerides (mmol/L)	0.24	0.004	−0.11	NS	−0.10	NS
GPx (U/L)	−0.17	0.043	−0.31	0.0002	−0.19	0.028
SOD (U/mg)	−0.20	0.015	−0.19	0.027	0.02	NS
Diabetes duration (years)	0.13	NS	0.31	0.0002	0.25	0.003

AOPP = advanced oxidation protein product; DNR = diabetic non-retinopathy; GPx = glutathione peroxidase; HbA_1c_ = glycated haemoglobin; NS = not significant; r = Pearson’s coefficient; SOD = superoxide dismutase; sRAGE = soluble form of RAGE.

**Table 3:** Linear correlation analysis between AOPP, pentosidine, sRAGE levels and several biochemical parameters in DR patients (*n* = 200).

Variables	AOPP (μmol/L)		Pentosidine (ng/mL)		sRAGE (pg/mL)		sRAGE/pentosidine ratio	
			
	*r*	*p* value	*r*	*p* value	*r*	*p* value	*r*	*p* value
HbA_1c_ (%)	0.08	NS	0.18	0.021	−0.10	NS	−0.23	0.003
Total cholesterol (mmol/L)	0.10	NS	0.05	NS	0.02	NS	−0.04	NS
Triglycerides (mmol/L)	0.30	0.0002	−0.10	NS	0.11	NS	−0.08	NS
GPx (U/L)	−0.35	<0.0001	−0.26	0.0006	−0.31	<0.0001	−0.05	NS
SOD (U/mg)	−0.16	0.033	−0.21	0.008	−0.03	NS	0.02	NS
Diabetes duration (years)	0.06	NS	0.18	0.013	0.15	NS	− 0.20	0.004
Retinopathy duration (years)	0.11	NS	0.12	NS	−0.15	0.043	0.48	0.010

AOPP = advanced oxidation protein product; DR = diabetic retinopathy; GPx = glutathione peroxidase; HbA_1c_ = glycated haemoglobin; NS = not significant; *r* = Pearson’s coefficient; SOD = superoxide dismutase; sRAGE = soluble form of RAGE.

**Table 4:** Risk factors for DR in type 2 DM patients (*n* = 371) by logistic regression.

Characteristics		Unadjusted model		Adjusted model a	
	
		OR (95% CI)	*p* value	OR (95% CI)	*p* value
Age (years)		0.98 (0.96–1.00)	0.022	0.96 (0.93–1.00)	0.052
*Gender*	Female	1.00 (reference)	–	–	–
	Male	0.87 (0.58–1.31)	0.511	–	–
*Race*	Malay	1.00 (reference)	–	–	–
	Chinese	1.30 (0.74–2.30)	0.365	–	–
	Indian	0.79 (0.50–1.23)	0.297	–	–
*Smoker*	No	1.00 (reference)	–	–	–
	Yes	0.51 (0.30–0.87)	0.013	0.40 (0.15–1.03)	0.057
*Hypertension*	No	1.00 (reference)	–	–	–
	Yes	0.79 (0.49–1.28)	0.347	–	–
BMI (kg/m^2^)		0.96 (0.92–1.01)	0.099	–	–
SBP (mmHg)		1.01 (1.00–1.02)	0.274	–	–
DBP (mmHg)		1.00 (0.98–1.02)	0.736	–	–
Total cholesterol (mmol/L)		1.25 (1.04–1.49)	0.018	1.46 (0.76–2.79)	0.251
Triglyceride (mmol/L)		1.25 (0.96–1.63)	0.100	–	–
HDL-C (mmol/L)		1.02 (0.53–1.95)	0.952	–	–
LDL-C (mmol/L)		1.25 (1.01–1.54)	0.045	0.68 (0.33–1.42)	0.308
ALT (IU/L)		1.00 (0.99–1.01)	0.998	–	–
AST (IU/L)		1.00 (0.99–1.02)	0.615	–	–
Diabetes duration (years)		1.08 (1.05–1.11)	0.000	1.08 (1.04–1.13)	0.000
HbA_1c_ (%)		1.34 (1.18–1.53)	0.000	1.20 (1.00–1.44)	0.046
GPx (U/L)		1.00 (1.00–1.01)	0.586	–	–
SOD (U/mg)		7.28 (4.42–17.57)	0.000	3.48 (0.55–22.18)	0.187
AOPP (μmol/L)		1.00 (1.00–1.01)	0.212	–	–
Pentosidine (ng/mL)		0.99 (0.97–1.01)	0.168	–	–
sRAGE (pg/mL)		1.00 (1.00–1.01)	0.090	–	–
sRAGE/pentosidine ratio		0.08 (0.02–0.37)	0.001	0.08 (0.01–0.98)	0.048

a Adjusted for age, gender and metabolic risk factors (BMI, HbA_1c_ and DM duration); ALT = alanine aminotransferase; AOPP = advanced oxidation protein product; AST = aspartate amino transferase; BMI = body mass index; CI = confidence interval; DBP = diastolic blood pressure; DNR = diabetic non-retinopathy; DR = diabetic retinopathy; GPx = glutathione peroxidase; HbA_1c_ = glycated haemoglobin; HDL-C = high-density lipoprotein cholesterol; LDL-C = low-density lipoprotein cholesterol; OR = odd ratio; SOD = superoxide dismutase; sRAGE = soluble form of RAGE; SBP = systolic blood pressure.
